# Questionnaire Background on the Hognose Snake (*Heterodon nasicus*) Breeders’ Awareness of the Risk of Being Bitten

**DOI:** 10.3390/ani11123537

**Published:** 2021-12-14

**Authors:** Damian Zieliński

**Affiliations:** Department of Animal Ethology and Wildlife Management, University of Life Sciences in Lublin, Akademicka St 13, 20-950 Lublin, Poland; damian.zielinski@up.lublin.pl

**Keywords:** *Heterodon nasicus*, hognose snake, envenoming, bite, survey

## Abstract

**Simple Summary:**

*Heterodon nasicus* is a popular pet reptile. It represents dipsadine colubrids, non-front-fanged snakes (NFFS) having Duvernoy’s glands situated in the temporal region, which deliver toxic saliva via modified dentition during swallowing even though they are generally not viewed as venomous animals. As shown by previous descriptions, handlers were bitten while feeding their snakes with bare hands or when the smell of food persisted on their hands after feeding. The results of a survey for hognose snake breeders showed that 47.7% (*n* = 41) of the respondents admitted that they had had a situation in which *H. nasicus* tried to bite them; it occurred during routine approaches to daily snake handling. These situations are definitely preventable using proper feeding methods and by avoidance of routine risky habits.

**Abstract:**

Several cases of *Heterodon nasicus* bites producing various symptoms have already been described. In this paper, a survey was conducted among Polish breeders of this species, where 47.7% of the respondents admitted that their snakes had tried to bite them, and 31.4% fed their snakes with their bare hands. When asked whether they feared being bitten by this snake during daily handling, an overwhelming majority of the respondents declared (74% of total negative responses, *n* = 63) that they were not afraid of being bitten. Based on the popularity of *H. nasicus* as a pet animal, it can only be assumed that the magnitude of accidental bites is much greater than that reported in published case reports. Therefore, it is important to try to educate pet breeders to report such cases and pay attention to the use of appropriate handling methods during contact with snake food and during feeding.

## 1. Introduction

The western hognose snake (*Heterodon nasicus*) is a very common species on the exotic pet market [1,2]. It represents dipsadine colubrids (Dipsadinae) [3], i.e., non-front-fanged snakes (NFFS) having Duvernoy’s glands situated in the temporal region [4]. Western hognose snakes are usually mild captives and do not bite in self-defense; therefore, they rarely bite humans when threatened and are generally not viewed as venomous [5]. While dipsadine colubrid snakes are generally considered harmless, many NFFS species have modified dentition delivering toxic saliva during the swallowing process [4,6].

There are few reports of western hognose snake bites, but the chief symptoms are edema, erythema, blister formation, and ecchymoses. The time to resolution of symptoms ranges from a few days to several months [5,7,8]. Past reports of bites are often poor in detail regarding the patient’s medical history or the course of recovery after the bite (e.g., no consultation with a physician after the bite); therefore, the duration of recovery of the bitten person should be predicted with caution. Despite the high popularity of this species as an exotic pet worldwide, only few bite reports have been published (Table 1 and [9]). The management of patients bitten by NFFS has already been described in detail [8,9,10]. However, further cases of bites should be reported to provide knowledge of the effects of snake bites depending on the circumstances and the physiological state of the patient.

In the case reports published to date, one of the most frequently described circumstances of *Heterodon* bites is the performance of daily activities in the terrarium, the feeding period, and the presence of the smell of food (mice, amphibians) on the hands during handling [6,9]. Therefore, it is potentially important to consider the awareness by owners and breeders of the possible risks associated with keeping *H. nasicus* in captivity.

In this study, the results of a questionnaire survey of hognose keepers’ awareness of the potential bite risk and consequences are presented.

## 2. Questionnaire Survey

### 2.1. Design of the Survey

Since most of the cases of bites by hognose snakes took place in captivity conditions, usually during everyday handling or feeding, a survey was conducted among Polish *H. nasicus* breeders and owners. The questionnaire was created using a Google form and shared on the Facebook group “*Heterodon*’s PL”. The survey was voluntary and anonymous and did not involve any financial gratification. In the survey, questions were asked about the number of animals owned. The other questions were as follows: Is the *Heterodon* a venomous animal? Has your *Heterodon* ever tried to bite you? Have you ever fed it with your bare hand (without using tweezers)? Other questions with answers assessed with the use of a 5-point scale (1-strongly not, 2-not, 3-not sure, 4-yes, 5-strongly yes) were also asked: Does the *Heterodon* pose any threat to humans? Do you fear being bitten by the *Heterodon* snake during daily handling? Three open-ended questions were asked: What precautions do you take to avoid being bitten? What would you do after being bitten by a *Heterodon*? Do you know the potential effects of a *Heterodon* bite? (multiple-choice question).

86 people responded to the survey.

### 2.2. Results of the Survey

**Figure 1:**
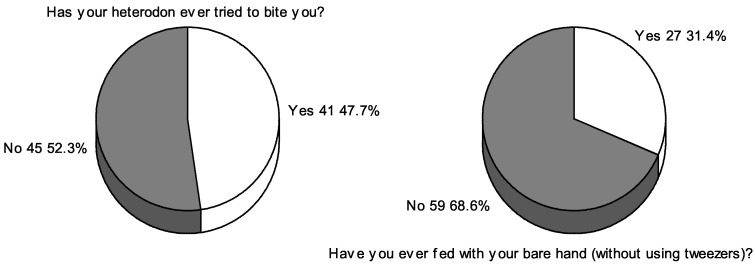
Respondents’ answers to questions related to bite incidents and applied feeding methods.

The vast majority of the respondents are owners of 1–3 *H. nasicus* snakes (66.3%, *n* = 57), and the rest of the respondents raise 4–15 individuals or more than 15 individuals of this species (19.8%, *n* = 17; 14%, *n* = 12, respectively). A total of 10.5% (*n* = 9) of the respondents were unaware that the species is venomous and may pose a threat to humans. More interestingly, 47.7% (*n* = 41) of the respondents admitted having had a situation in which their *H. nasicus* snake tried to bite them (Figure 1). They were not asked for details (whether these were actual bites or just attempts to scare them away), but the scale of these responses shows that, despite the small number of scientific reports of bites by *H. nasicus*, there may be many more such unreported cases all around the world. In response to the question of whether they sometimes serve food with their bare hands, 31.4% (*n* = 27) of the respondents confirmed hand-feeding their hognose snakes (Figure 1). Since hognose snakes are most often encouraged to bite by feeding without the use of tweezers or the presence of food odors (mice, amphibians) on the hands, 31.4% of the respondents are potential victims of bites that may occur during feeding. These bites can be avoided in the vast majority of cases by applying proper feeding methods—leaving food inside the container or feeding with the use of tweezers as well as careful hand hygiene after handling food items.

**Figure 2:**
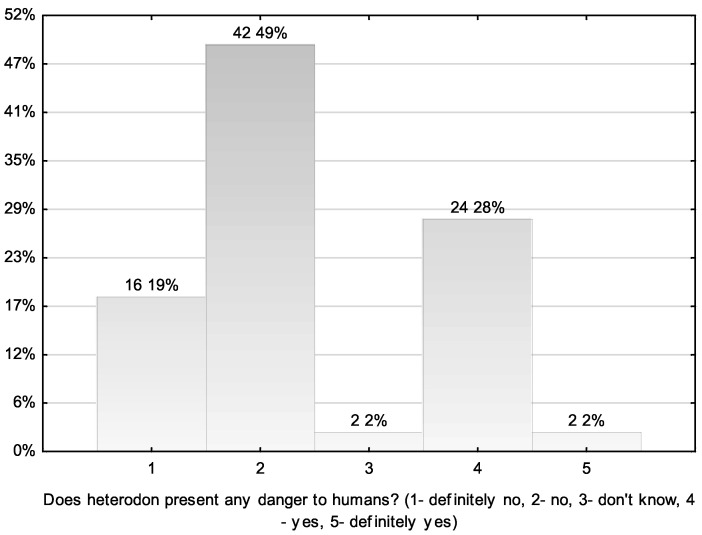
Respondents’ answers to the question of a potential threat posed by *Heterodon* snakes to humans (*n*; %).

**Figure 3:**
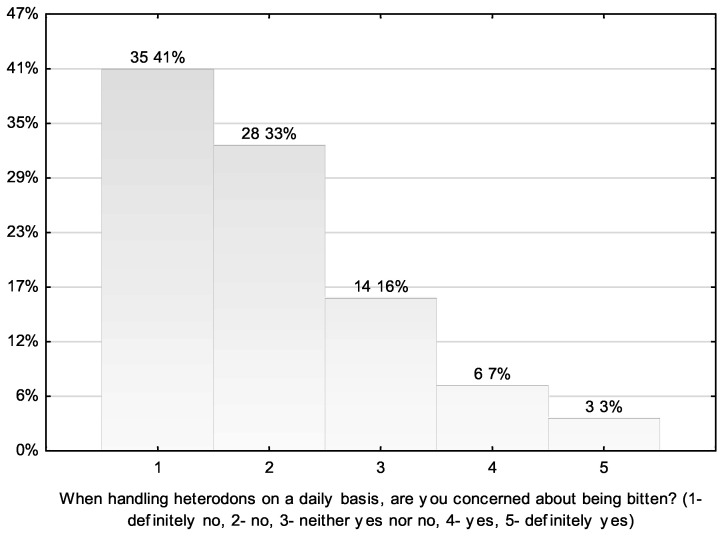
Respondents’ answers to the question about their fear of being bitten by *H. nasicus* during daily handling (*n*; %).

Another interesting topic is the awareness of the hognose snake owners that this species poses a threat to humans (risk of medically important bites) and their fear of being bitten by the snakes during daily handling. In general, the respondents rated the threat from this snake species as insignificant (Figure 2), with 68% choosing the answer “definitely not” and “not” on the response scale (19%, *n* = 16; 49%, *n* = 42, respectively). Another 28% (*n* = 24) of the respondents chose the answer “yes”. In contrast, when asked about their fear of being bitten by *H. nasicus* during daily handling, an overwhelming majority of the respondents declared (74% of total negative responses, *n* = 63) that they were not afraid (Figure 3). Only 16% (*n* = 14) were undecided (neither yes nor no) about their answer to this question.

The most common precautions taken by the respondents to avoid being bitten were as follows: using tweezers during feeding, avoiding contact of hand skin with food, frequent hand washing during feeding (if one has many snakes), using an ophiological hook to relocate the snake, knowledge and ability to interpret the behavior of the species, and training snakes using classical conditioning so they do not associate handling with food. Only a few respondents do not use any precautions in handling this species on a daily basis.

In the next question, the respondents were asked to describe their reactions and actions taken after *H. nasicus* bites (under the hypothetical assumption that there was a serious bite). Only 3.5% (*n* = 3) would seek medical help first, 75.6% (*n* = 65) would postpone calling for help until symptoms worsen, and 7% (*n* = 6) claim they would be fine without medical help. The rest of the respondents described that their next practice (remedies) consisted in taking antihistamines or calcium and observation of the bite site. Several respondents also reported their fear of calling for medical help due to the lack of knowledge of hognose snakes bites in Poland. In the last question (multiple choice), the respondents were asked to mark symptoms that may occur after being bitten by a western hognose snake. As many as 97.7% (*n* = 84) of the respondents indicated swelling at the site of the bite, and 81.4% (*n* = 70) indicated pain. The next two symptoms which were mostly chosen were tenderness and anaphylactic shock (61.6, *n* = 53; 58.1%, *n* = 50, respectively). Other symptoms which were often pointed out were dizziness, pruritus, nausea, feeling of burning, fever, blistering, and bleeding (answer range of 31.4–48.8%). It is worth mentioning that some of the owners indicated differences in body reactions depending on the site of the bite, the duration of attachment of the snake to the body, and the degree of sensitization to the venom (e.g., in the case of bee venom; the reaction may range from slight swelling to anaphylactic shock). It should be noted that the respondents’ statements are subjective, based on their assumptions (non-medical) as to the consequences of being bitten by a hognose snake.

## 3. Discussion

Previous case reports of bites by this snake species have reported symptoms such as edema around the bite site, erythema, wound discharge, bleeding, pruritus, burning, tenderness, formation of ecchymoses, blistering, thrombocytopenia, and generally pain and nausea [5,9,13] (see Table 1). The thrombocytopenia mentioned here is the only described case of a systemic reaction following a hognose snake bite [13]. Here, only swelling and some pain reactions were observed by the bitten persons. However, the time of total recovery may vary from days to months. There are still too few formally reported hognose snakes bites to fully determine the time required for full recovery, especially adequately described medical cases. The recovery time may also be dependent on the length of attachment of the snake to the body. In other words, it is dependent on the duration of the bite and insertion of venom into the victim’s wound. Victims often described that their attempts to detach the snake provoked it to bite even deeper into the body, thus increasing the risk of injecting large amounts of venom [6,9]. To unclench the jaws of the snake, they usually used force or put some object (such as a pencil) between the jaws and the body, which usually relatively quickly gave the desired result. Another method mentioned in publications is to place a biting snake under cold running water. Unfortunately, it is usually not effective or takes too much time for the snake to release the grip [6].

It is worth noting that, in many cases, the bite occurred as a result of the smell of a mouse on the hand with which the snake was later grabbed. Unfortunately, the routine practice in everyday snake handling in captivity can be a common cause of “accidental” (not intentional) bites in which the snake mistakes the human body for food (based solely on the scent) [6,15]. As early as 1960, Bragg described his case of a *Heterodon* bite that resulted from snake handling after his hand had previously come into contact with dead frogs [16].

As indicated by the results of the survey, although the majority of the respondents are not afraid of being bitten by their *Heterodon* snakes, they do use various methods of prevention, including avoiding hand feeding and frequent hand washing to avoid the scent of rodents. However, many owners are still used to feeding snakes with their bare hands. Therefore, more attention should be paid to dissemination of information among breeders of this species on the risks posed by such practices.

Some limitations of this paper must be emphasized. First, this was an online questionnaire; therefore, the sample was not representative because the respondents decided whether to take part in the survey or not. The survey was placed only in one Facebook group, which potentially limited the number of the respondents. The total number of the surveyed hognose snake owners was low (*n* = 86), which is insufficient for reliable statistical analysis. However, this is an inherent problem in most survey research related to a small research group (here: hognose snake owners), and the result of the survey itself certainly is important for describing the problem of potentially dangerous bites from *H. nasicus* in captivity. Secondly, the survey used questions about potentially dangerous situations, which may have resulted in attempts by some respondents to conceal such cases. Additionally, when asked about potential consequences resulting from a hognose snake bite, the respondents expressed their assumptions. These responses should be regarded as having low quality due to the lack of medical knowledge of the respondents, their subjective opinions, often referring to knowledge passed (often erroneously) from keeper to keeper, and suggesting that the use of medications (remedies) is generally considered appropriate in the case of bites by venomous snakes.

## 4. Conclusions

Based on the popularity of *H. nasicus* as a pet animal, it can only be assumed that the magnitude of this phenomenon (accidental bites) is much greater than in the number of published case reports. Therefore, it is important to educate pet breeders to report such cases and to pay attention to the use of appropriate handling methods during contact with snake food and during feeding.

## Figures and Tables

**Table 1:** Previously * reported cases of *Heterodon nasicus* bites with detailed information about symptoms, the origin of the snake, bite site, and circumstances.

Size or Age of the Snake (cm, yo)	Age of Victim (Years), Sex	Origin of the Snake **	Bite Site, Circumstances	Symptoms	Aid by Professional	Time to Resolution of Symptoms	Reference
ND	26, male	Wild-caught (during the study in a lab)	Middle finger (left hand), accidentally	P, B, T, E	No	1 week	Kroll (1976) [11]
60	38, male	Wild-caught (caught in 1990, bitten in 2006 in captivity)	Fifth finger (left hand), during daily routine	B, P, T, E	No	4 days	Averill-Murray (2006) [6]
45	21, female	Captive	Arm near the elbow, during feeding	E, EC, mild P, T, L, B, ER	Yes	5 months	Weinstein and Keyler (2009) [9]
3 yo	26, male	Captive	Left hand, accidental related to post-feeding manipulation inside the snake enclosure (scent of a mouse on hand)	E, mild P, B, S, B	Yes	5 months (50% less mobility of the bitten finger)	Flenghi et al. (2018) [12]
2 yo	20, female	Captive	Left hand, the scent of a mouse during handling	P, T, E, EC, B, DC, TC	Yes	4 months	Brandehof et al. (2019) [13]
ND	19, male	Captive	Left arm, during feeding	E, EC, B, ER, P	Yes	2 months	Kato et al. (2019) [5]
ND	ND, female	Captive	Left antecubital fossa, during feeding	E, B, EC, P, S	Yes	“several months“	Weinstein et al. (2011) [4]
ND	ND, male	Captive	Middle finger of left hand (proximal phalanx of digit), ND	E, B, EC	Yes	ND	Weinstein et al. (2011) [4]

Abbreviations: B—blistering, DC—skin discoloration, E—edema, EC—ecchymoses, ER—erythema, L—lymphadenopathy, P—pain, S—stiffness, T—tenderness, TC—thrombocytopenia, ND—no data. * Some of the cases are not included in the table because of the paucity of detail or very distant dates (relying, for example, on McKinstry 1978 [14], where he cites studies from 1881 to 1974, in which, mainly in the course of seeking answers to the question of *Heterodon* venom, researchers deliberately allowed these snakes to bite them). For other *Heterodon* bites records summary see Weinstein and Keyler (2009) [9]. ** Wild—snake encountered in the wild, wild-caught—wild snake caught and kept in a captive environment, captive—captive born snake kept in a captive environment.

## Data Availability

The data presented in this study are available on request from the corresponding author.
